# Assessment of changes in the *brca2* and *p53* genes in breast invasive ductal carcinoma in northeast Brazil

**DOI:** 10.1186/0717-6287-47-3

**Published:** 2014-03-26

**Authors:** Eduardo AVF Ramalho, João LQ Silva-Filho, Marina FS Cartaxo, Carmelita BL Cavalcanti, Moacyr JBM Rêgo, Maria BM Oliveira, Eduardo IC Beltrão

**Affiliations:** Keizo Asami Immunopathology Laboratory, Federal University of Pernambuco, Recife, Pernambuco Brazil; Biochemistry Department, Federal University of Pernambuco, Recife, Pernambuco Brazil; Laboratório de Imunopatologia Keizo Asami (LIKA), Universidade Federal de Pernambuco (UFPE), Av. Prof. Moraes Rego, s/n, Cidade Universitária, Recife, PE 50670-901 Brazil

**Keywords:** Breast cancer, Epigenetics, Metastasis, Methylation, Polymorphism

## Abstract

**Background:**

BRCA protein interacts with at least 13 different proteins that have been implicated with cancer susceptibility and loss of BRCA function is correlated to sensitivity to DNA crosslinking agents in preclinical models.

**Results:**

BRCA2 methylation frequency was 44%, p53 Pro22 allele frequency was 32% and heterozygous frequency of Arg/Pro72 genotype was 60% which could be associated as risk factor for metastasis (*p* = 0.046 OR = 4.190). Regarding to polymorphism of codon 249 the frequency of Arg249 allele presented 82% which was considered not statistically significant.

**Conclusions:**

There was not statistical significance to BRCA2 promoter methylation with any parameters chosen. However, our findings suggest that patients who present heterozygous genotype at codon 72 of p53 gene may have a major susceptibility to any type of metastasis and this could serve as potential auxiliary biomarker for poor prognosis.

## Background

It is well known that BRCA2 gene encode functionally related proteins that play critical roles in DNA double-strand breaks repair [1–3]. BRCA protein interacts with at least 13 different proteins that have been implicated with cancer susceptibility, suggesting that BRCA gene works as an essential signaling network dedicated to genome integrity [4–8]. Loss of BRCA function results in development of chromosomal instability and this ‘BRCAness’ (loss of BRCA function or BRCA-null) phenotype correlated to sensitivity to DNA cross-linking agents in preclinical models [9–11].

In contrast to non-coding regions of the genome where most CpGs are methylated, CpG islands in 5′ cis-regulatory regions of genes are usually unmethylated. Methylation of these CpG islands during the development or disease processes is associated with post-translational histone modifications that lead to a locally condensed inactive chromatin structure and gene silencing [12, 13]. During tumorigenesis, there is a progressive loss of global DNA methylation and at the same time regional hypermethylation [14]. Tumor-specific hypermethylation of CpG islands in 5′ promoters can inactivate genes for DNA repair, cell cycle control and other mechanisms that prevent neoplastic transformation in a normal cell [15]. Epigenetic abnormalities do not only occur as secondary changes at all stages of tumor evolution, but can also act as initiating events [16].

As a diagnostic technique methylation-specific PCR (MSP) could be highlighted [17]. The precise mapping of DNA methylation patterns in CpG islands has become essential for understanding many biological processes such as gene regulation, X chromosome inactivation and silencing of tumor suppressor genes. The MSP technique can quickly assess the methylation pattern of virtually any promoter region which contains therein one or more CpG islands. MSP is sensitive to 0.1% methylated alleles of a particular CpG island and can be also performed in formalin-fixed paraffin-embedded tissues the most common sample presentation for diagnosis. Early detection of aberrant methylation in carcinogenesis related genes may be essential for diagnosis, prognosis and/or detection of metastatic potential [17].

DNA methylation is an important epigenetic mechanism that occurs in CpG sites and is directly involved in gene regulation [18–22]. In human cancer, such non-genetic modification which can be heritable consists in a powerful mechanism responsible for the inhibition of different genes, including tumor suppressor genes [23].

The tumor suppressor gene p53 plays a major role in cell cycle control, apoptosis and maintenance of DNA integrity. Due to its importance in cell cycle control and integrity, it was nicknamed “genome guardian” [24, 25]. Mutations and genetic polymorphisms may alter the function of p53 proteins leading to imbalances in the major gene functions [26, 27].

Among TP53 gene polymorphisms, the most studied is the G to C transversion in exon 4 at codon 72 (rs1042522), which encodes two distinct functional allelic forms, arginine (Arg) and proline (Pro) and results in three distinct genotypes, Arg / Arg, Pro / Pro and Arg / Pro, each one encoding different p53 isoforms [28].

Another hotspot in p53 gene is frequently founded at exon 7 which occurs a G to T transversion at the third position of codon 249 (rs28934571) of the coding sequence of the gene which results in the substitution of Serine for Arginine [29]. This study analyzed BRCA2 promoter region methylation pattern and p53 Single Nucleotide Polymorphisms (SNP) correlating with clinic-pathological parameters such as age, tumor size, lymph node involvement and metastasis of patients from Recife, Pernambuco, Northeast Brazil.

## Results

### BRCA2 MS-PCR

**Figure 1:**
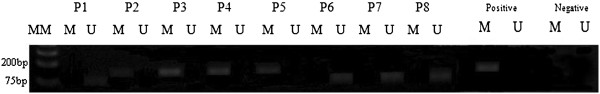
**1% Agarose gel showing methylation status of promoter BRCA2 gene in IDC patients.** MM: Molecular Marker (Fermentas GeneRuler 1 kb Plus); P: IDC Patients (1–8); M: Methylated alleles (139 bp); U: Unmethylated alleles (79 bp). Positive control: DNA from human blood treated by *Sss*I Methylase; Negative control: Nucelase free water (blank control).

DNA samples were extracted, quantified and subsequently amplified by PCR using the housekeeping gene β-globin (data not shown) from fifty biopsies from patients diagnosed with IDC and five healthy controls were subjected to MS-PCR amplification. The frequency of BRCA2 promoter methylation in all IDC patients corresponded to 44% (22 of 50) (Figure 1).

### p53 PCR-RFLP

**Figure 2:**
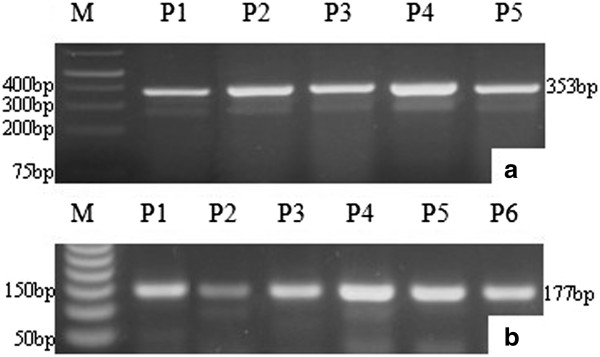
**PCR-RFLP amplified products of p53 gene. a**: amplification of p53 codon 72 (353 bp) M: Molecular Marker (Fermentas GeneRuler 1 kb Plus); **b**: Amplified products of p53 codon 249 (177 bp) M: Molecular Marker (Promega, 50 bp); P: Patients.

**Figure 3:**
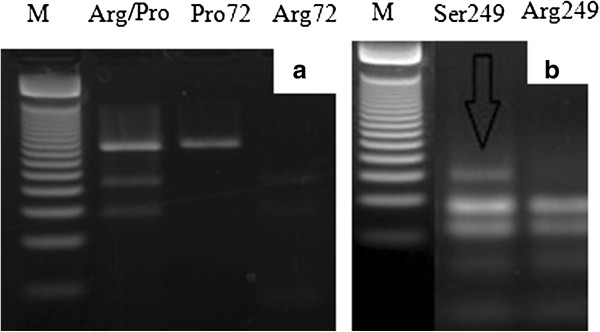
**PCR-RFLP analysis of p53 gene (Arg/Pro genotype). a**: p53 codon 72. M: Molecular weight marker (Promega 50 bp). Heterozygous genotype Arg/Pro; Proline genotype showing lack of restriction site (353 pb band); Arginine genotype cleaved by the enzyme resulting in two fragments (214 and 139 bp). **b**: p53 codon 249. M: Molecular weight marker (Promega 50 bp); Arrow indicates the fragment corresponding to 249Ser genotype (154 pb) not cleaved by HaeIII; Enzyme digestion fragments showing the Arg249 genotype with 92 and 62 bp.

Amplified products of exon 4 and exon 7 were 353 bp and 177 bp, respectively (Figure 2a and b). Detection of p53 codon 72 polymorphisms by PCR-RFLP was successfully conducted in all cases and controls. Arginine allele was cleaved by B*st*UI yielding two smaller fragments (214pb and 139pb). On the other hand, Proline allele resulted in a single band of 353 bp. Heterozygous samples showed a three bands genotype (353, 214 and 139pb) as shown in Figure 3a. PCR-RFLP of p53 codon 249 was successfully conducted in all cases and controls. Arg249 digestion presented four fragments (92, 62, 23 and 12pb). Serine249 genotype showed loss of restriction site for H*ae*III yielding an uncleaved fragment of 154 bp besides 92, 62, 23 and 12 bp fragments (Figure 3a and b).

The genotypic frequency of Proline72 allele was 32% (16 of 50). Unlike Arg/Pro72 genotype which presented a frequency of 60% (30 of 50), Arg72 frequency was only 8% (4 of 50). In relation to allelic frequency, our results revealed a predominance of Pro72 allele with 62% while for Arg72 allele the frequency was 38%. Comparison between p53 codon 72 genotype and clinical pathologic data is summarized in Tables 1 and 2. p53 codon 249 analysis has shown no statistic significance with any parameters chosen in this study and its genotyping frequency of Serine249 allele was 18% (9 of 50) while Arg249 allele was 82% (41 of 50). We also tried to achieve some association between BCRA2 methylation status and p53 polymorphisms of IDC patients analyzing each patient who presented amplified methylated alleles and correlate it with any possible p53 gene isoform expressed. However, nothing significant was founded (*p* > 0.05).

**Table 1:** **Homozygote Pro/Pro72** ***versus*** **Heterozygote Arg/Pro72**

Parameter	Codon 72 polymorphism		***p***	RR (IC95%)	OR (IC95%)	χ^2^
	Pro/Pro72	Arg/Pro72				
	(n = 15)	(n = 30)				
**Age (in years)**						
30–50	6	13	0.903	0.9474 (0.3965 to 2.263)	0.9231 (0.2548 to 3.344)	0.0148
>51	8	16				
**Tumor size (cm)**						
<5	11	26	0.2701	0.5946 (0.2536 to 1.394)	0.4231 (0.08932 to 2.004)	1.216
>5	4	4				
**Lymph node involvement**						
Negative	7	10	0.3845	1.441 (0.6372 to 3.260)	1.750 (0.4928 to 6.215)	0.7563
Positive	8	20				
**Metastasis**						
Negative	11	14	**0.046**	2.787 (0.9011 to 8.617)	**4.190** (0.9686 to 18.13)	3.960
Positive	3	16				

Arg (Arginine); Pro (Proline).

**Table 2:** **Homozygote Arg/arg72** ***versus*** **Homozygote Pro/pro72**

Parameter	Codon 72 Polymorphism		***p***	RR (IC95%)	OR (IC95%)	χ^2^
	Arg/Arg72	Pro/Pro72				
	(n = 4)	(n = 15)				
**Age (in years)**						
30–50	2	6	0.7998	1.25 (0.2225 to 7.022)	1.333 (0.1436 to 12.38)	0.0642
>51	2	8				
**Tumor size (cm)**						
<5	2	11	0.3724	0.4615 (0.08389 to 2.539)	0.3636 (0.03756 to 3.520)	0.7957
>5	2	4				
**Lymph node involvement**						
Negative	0	7	0.0856	0	0.125 (0.005770 to 2.748)	2.956
Positive	4	8				
**Metastasis**						
Negative	0	11	**0.004**	0	**0.0338** (0.0014 to 0.7955)	8.082
Positive	4	3				

Arg (Arginine); Pro (Proline).

When analyzing methylation status and p53 hotspots, among all patients who presented BRCA2 promoter methylation, 68.2% expressed Arg / Pro 72 genotype and Pro / Pro genotype frequency was 31.8%. There was no Arg / Arg homozygote genotype frequency in patients who had BRCA2 promoter methylation. Concerning codon 249 polymorphisms, the frequency of Arginine 249 was 81.8% for methylation BRCA2 patients, while for Serine 249 was 18.2%. However no correlation between methylation status/polymorphism and clinical-pathological data could be established with any parameter chosen in this study.

## Discussion

In this study, we aimed to understand human TP53 codon 72 polymorphism associated with IDC development since this common substitution of one base pair at codon 72 of the gene changes the biochemical and functional properties of the protein. The frequency, time and spectrum of p53 gene mutation may be useful to provide clues to the etiology and pathogenesis of human cancer. Several studies proposed the role of codon 72 polymorphism as a risk factor for different types of cancers such as stomach, lung and bladder [30]. Among three codon 72 genotypes, Arginine is more susceptible to degradation by human papillomavirus (HPV) E6 type-18 protein and suppresses cellular transformation more effectively than Pro72. On the other hand Arg72 is more efficient than Pro72 to induce apoptosis [27]. Additionally, variants of the TP53 gene seem to confer differential responses to chemotherapy [31]. The Arg / Arg genotype was reported to be associated to a higher response rates and survival in patients with breast [32, 33], lung [34] or head and neck cancer [35].

Our results showed a predominance of the proline allele (62%) and the heterozygous Arg / Pro represents the most common genotype (60%) among this research subjects. These findings are not in line with a recent study of TP53 polymorphism among 96 individuals [36] from a Brazilian population where was found a high prevalence of Arginine allele (68%) showing a contrast to our study where Arginine allele frequency was 38% (19 of 50). However, we did not find an association between p53 genotype and breast cancer development.

Hence, our findings showed a low prevalence of homozygosity for Arginine 72 alleles in patients with breast cancer in opposition to results found in cases from Greece [37], Turkey [38] and in Southern Brazil [26]. These findings may be result of a high heterogeneity in Northeast Brazilian population mainly as consequence of the colonization history (Europeans, Africans and Brazilian Indians).

In most cases, when trying to establish some association with genotypic or allele polymorphism the disease presents an unfavorable prognosis for the patient. Although our results demonstrate a 60% genotypic frequency related to Arg / Pro72, no association with IDC development could be established. However, these data suggest a relative association between heterozygous Arg / Pro genotype and metastasis (*p* = 0.046 OR = 4.190) which may be related by increasing the chance to develop a future metastasis since this feature was observed in patients with a more aggressive phenotype, being more genetically unstable and consequently more susceptible for metastasis. These findings could be useful in the near future where we may be able to promote prevention campaigns directed to the people’s genotype since the Pro72 genotype presented itself as more stable against any type of metastasis (*p* = 0.004 OR = 0.0338).

Opposite results were found regarding the involvement of Arg / Pro heterozygous variant and increased breast cancer risk in North Indian population [26]. Another study reported that Proline homozygosity at p53 codon 72 is associated with decreased breast cancer risk in Arab women [39].

So far, there are no results that can support any proposed theory about the role of codon 72 polymorphisms as risk factor for breast cancer, this may be associated to a large number of studies showing conflicting results. Our analysis concerning exon 7 showed a frequency of 18% for Ser249 allele and 82% for Arg249 of breast cancer patients, which was considered not statistically significant. Both genotypes related to codon 249 polymorphisms showed no significance when crossed with any parameters chosen for this study. Our results are in accordance with studies in Indian population showing that codon 249 polymorphisms had no association as risk factor for breast cancer [25].

There are no doubts about the importance of methylation pattern in BRCA2 promoter region in breast and ovarian carcinoma. Our findings demonstrate a frequency of 44% methylation in promoter region of BRCA2. Comparison between BRCA2 methylation status and p53 polymorphisms, Arg249 was the most prevalent (81.8%) genotype among patients who presented promoter methylation in BRCA2. Therefore, no association between methylation status and p53 polymorphisms could be established.

BRCA2 and p53 genes being extensively studied worldwide, there are some medical centers performing genetic tests for evaluation of germline mutations in BRCA2 for genetic counseling, but little is known about the epigenetic profile if would be a useful auxiliary tool in early diagnosis of breast and ovarian cancer. Further studies will be necessary to establish methylation status of tumor suppressor genes as early diagnosis tool in cancer development. In this sense, our findings suggest that the northeast Brazilian patients who present heterozygous genotype Arg/Pro at codon 72 of p53 gene may have a major susceptibility to develop any type of future metastasis which could be indicate as potential auxiliary biomarker for poor IDC prognosis.

## Conclusion

Our findings suggest that patients who present heterozygous genotype at codon 72 of p53 gene may have a major susceptibility to any type of metastasis and this could serve as potential auxiliary biomarker for poor prognosis.

## Methods

### Samples

Fifty formalin-fixed and paraffin-embedded biopsies diagnosed as invasive ductal carcinoma (IDC) and five normal tissues (from reducing mastoplasty) were obtained from Anatomy Pathology Service of Hospital das Clínicas at Federal University of Pernambuco (UFPE), Brazil. This study was approved by the Health Science Center Bioethical Board of UFPE (SISNEP FR – 272931, CEP/CCS/UFPE No 195/09). Exclusion criteria included patients under 30 year-old and samples with different types of carcinoma besides IDC. Clinical and pathological parameters such as: age, tumor size, lymph node invasion and metastasis were evaluated.

### DNA isolation

After repeated attempts to standardize the whole method, was stipulated that ten sections (10 × 2 μm) of each FFPE biopsy would be necessary to be placed into an Eppendorf tube (2 mL) for deparaffinization. Xylene (1 mL) was added, mixed (40–50 seconds) and samples incubated at 25°C for 30 min (vortexed every 10 min). Samples were centrifuged at 14,000 rpm for 3 min and xylene was discarded. Ethanol (1 mL) was added and mixed by inversion followed by centrifugation at 14,000 rpm for 3 min. Ethanol was removed and ethanol/centrifuge process was repeated. The supernatant was discarded and the samples were dried using a vacuum centrifuge. After drying, samples received 400 μL of cell lysis buffer (0.5 M EDTA, 5 M NaCl, 1 M Tris), 36 μL of SDS (20x), 24 μL of Proteinase K (20 mg/mL) and 20 μL of MilliQ water. Samples were incubated at 65°C in a water bath for 18 h. After that 420 μL of 5 M NaCl solution was added and samples were centrifuged at 14,000 rpm for 20 min. Supernatant was transferred to an Eppendorf tube, and 800 μL of cold isopropyl alcohol was added followed by centrifugation at 14,000 rpm for 20 min. Supernatant was discarded and ethanol (500 μL) was added and briefly vortexed. Samples were centrifuged at 14,000 rpm for 15 min and the supernatant was discarded. DNA was vacuum centrifuge dried, dissolved in 100 μL of TE buffer and stored at −20°C until use. DNA quantification was performed by Nanodrop 2000 Spectrophotometer (Thermo Scientific, USA) and the amount of DNA was approximately 50 ng/μl for each sample.

### Identification of CpG islands in the promoter region of BRCA2 gene

Methyl Primer Express® software (Applied Biosystems) was used in order to identify CpG islands and design primers for MSP technique. Methylation specific primers were designed to the promoter region in exon 1 in the 5′ untranslated region of the BRCA2 gene. Primers for amplification were as follows: *BRCA2* Methylated Forward (5′- AAATTAGGCGGTAGAGGC-3′), and Reverse (5′- ATAAACTAACAAAAACCGCG-3′), *BRCA2* Unmethylated Forward (5′- TTGAAATTAGGTGGTAGAGGT-3′) and Reverse (5′- AAATAAACTAACAAAAACCACAC-3′).

### Bisulfite treatment

Bisulfite treatment of genomic DNA (2 μg) was carried out using Epitect Bissulfite Kit (QIAGEN) following manufacturer’s instructions.

### BRCA2 Methylation analysis (MSP)

All MSP reactions were performed using GoTaq® Green Master Mix (Promega) following the manufacturer’s instructions. It was used 0.4 μM of each primer and 50 ng of DNA template (final volume reaction was 12.5 μL). Amplification conditions were: BRCA2 methylated allele (hot start at 94°C for 5 min followed by 40 cycles of 94°C for 50 sec, 51°C for 40 sec and 72°C for 45 sec); BRCA2 unmethylated alleles (hot start at 94°C for 5 min followed by 35 cycles of 94°C for 45 sec, 50°C for 40 sec and 72°C for 45 sec). In all MSP reactions was performed a 5 min final extension. Reaction products were separated by electrophoresis on 1% agarose/Sodium borate gel at 100 V for 90 minutes, stained with ethidium bromide (0.5 μg/mL) and photodocumentated in LPIX (Loccus Biotechnology). For MSP technique was expected a 139 bp amplicons to methylated alleles and 79 bp for unmethylated alleles. As a control for the methylated-specific primers, *Sss*I methylase-treated DNA was used to generate a full methylated DNA at all of the CpG sites. Water was used as template in negative control.

### p53 genotyping

All PCR reactions were performed using GoTaq® Green Master Mix (Promega) following the manufacturer’s instructions. Primers were obtained according to IARC TP53 Database where exon 4 was amplified using 0.4 μM of each primer: forward (5′-TGCTCTTTTCACCCATCTAC-3′) and reverse (5′-ATACGGCCAGGCATTGAAGT-3′) and for exon 7 it was used 0.4 μM of each primer: forward (5′-AGGCGCACTGGCCTCATCTT-3′) and reverse (5′-TGTGCAGGGTGGCAAGTGGC-3′). In both reactions it was used 50 ng of DNA template (final reaction volume = 12.5 μL). The expected amplified products were 353 bp for exon 4 and 177 bp for the exon 7. Amplicons were evaluated on 1% agarose gel electrophoresis and stained with ethidium bromide (0.5 μg/mL). Amplification conditions were hot start at 94°C for 2 min followed by 40 cycles of 94°C for 1 min, 60°C for 45 sec and 72°C for 45 sec with a final extension at 72°C for 5 min. As negative control, a sample without DNA template was also included in the PCR reaction to ensure that no contamination was introduced.

PCR data was confirmed by performing RFLP analysis. It was used 0.5 μL (10 units) of enzyme B*st*UI (Biolabs), 2 μL of 1x buffer, 15 μL of DNA fragment and 2.5 μL of nuclease free water (total volume of 20 μL). Reactions were carried out for 2 h at 60°C in the thermocycler. Restriction products were electrophoresed on 2% agarose gel at 90 V for 120 minutes and stained with ethidium bromide (0.5 μg/mL). The 177 pb fragment derived from exon 7 of the p53 gene was digested using 0.5 μL (5 units) of the H*ae*III (Biolabs), 2 μL of 10x buffer, 15 μL of DNA fragment and 2.5 μL of nuclease free water (total volume of 20 μL). Reactions were developed for 2 h at 37°C followed for 20 min at 80°C for enzyme activity inhibition. Resulting fragments were evaluated on a 2% agarose gel at 90 V for 120 minutes and stained with ethidium bromide (0.5 μg/mL). The mutation studied on p53 codon 72 was rs. 1042522 and on codon 249 of p53 was AGG → AGT a substitution in the third position of the codon changing an arginine for a serine.

### Statistical analysis

All Statistical analysis was performed using GraphPad Prism version 5. The contingency analysis was used to compare the associations of categorical variables and *p* values were derived from the method of chi-square. The association between TP53 codon 72 polymorphism and clinic parameters was estimated by calculating the odds ratio (OR) and its 95% confidence interval (CI). OR estimate the chance of an event occurring in one group compared to another group. *p* < 0.05 was considered statistically significant.

## References

[1] Venkitaraman AR (2009). Linking the cellular functions of BRCA genes to cancer pathogenesis and treatment. Annu Rev Pathol.

[2] Tutt A, Ashworth A (2002). The relationship between the roles of BRCA genes in DNA repair and cancer predisposition. Trends Mol Med.

[3] Karran P (2000). DNA double strand break repair in mammalian cells. Curr Opin Gen Dev.

[4] Solyom S (2012). Breast cancer-associated Abraxas mutation disrupts nuclear localization and DNA damage response functions. Sci Transl Med.

[5] Erkko H, Xia B, Nikkilä J, Schleutker J, Syrjäkoski K, Mannermaa A, Kallioniemi A, Pylkäs K, Karppinen SM, Rapakko K, Miron A, Sheng Q, Li G, Mattila H, Bell DW, Haber DA, Grip M, Reiman M, Jukkola-Vuorinen A, Mustonen A, Kere J, Aaltonen LA, Kosma VM, Kataja V, Soini Y, Drapkin RI, Livingston DM, Winqvist R (2007). A recurrent mutation in PALB2 in Finnish cancer families. Nature.

[6] Rahman N, Seal S, Thompson D, Kelly P, Renwick A, Elliott A, Reid S, Spanova K, Barfoot R, Chagtai T, Jayatilake H, McGuffog L, Hanks S, Evans DG, Eccles D, Easton DF, Stratton MR, Breast Cancer Susceptibility Collaboration (UK) (2007). PALB2, which encodes a BRCA2-interacting protein, is a breast cancer susceptibility gene. Nat Genet.

[7] Seal S, Thompson D, Renwick D, Elliott A, Kelly P, Barfoot R, Chagtai T, Jayatilake H, Ahmed M, Spanova K, North B, Mcguffog L, Evans DG, Eccles D, Easton DF, Stratton MR, Rahman N, The Breast Cancer Susceptibility Collaboration (UK) (2006). Truncating mutations in the Fanconi anemia J gene BRIP1 are low-penetrance breast cancer susceptibility alleles. Nat Genet.

[8] Nikkilä J, Coleman KA, Morrissey D, Pylkäs K, Erkko H, Messick TE, Karppinen SM, Amelina A, Winqvist R, Greenberg RA (2009). Familial breast cancer screening reveals an alteration in the RAP80 UIM domain that impairs DNA damage response function. Oncogene.

[9] Tassone P, Di Martino MT, Ventura M (2012). Loss of BRCA1 function increases the antitumor activity of cisplatin against human breast cancer xenografts in vivo. Cancer Biol.

[10] Powell SN, Kachnic LA (2008). Therapeutic exploitation of tumor cell defects in homologous recombination. Anticancer Agents Med Chem.

[11] Chirnomas D, Taniguchi T, De la Vega M (2006). Chemosensitization to cisplatin by inhibitors of the Fanconi anemia/BRCA pathway. Mol Cancer Ther.

[12] Nygren AOH, Najim A (2005). Methylation-Specific MLPA (MS-MLPA): simultaneous detection of CpG methylation and copy number changes of up to 40 sequences. Nucleic Acids Res.

[13] Esteller M (2005). Aberrant DNA Methylation as a cancer-inducing mechanism. An Rev Pharmacol Toxicol.

[14] Momparler RL (2003). Cancer epigenetics. Oncogene.

[15] Bird AP (1986). CpG-rich islands and the function of DNA methylation. Nature.

[16] Larsen F, Gundersen G, Prydz H (1992). Choice of enzymes for mapping based on CpG islands in the human genome. Genet Anal Tech Appl.

[17] Herman JG, Graff JR, Myöhänen S, Nelkin BD, Baylin SB (1996). Methylation-specific PCR: a novel PCR assay for methylation status of CpG islands. Proc Natl Acad Sci.

[18] Gomez-Lazaro M, Fernandez-Gomez FJ, Jordán JJ (2004). p53: twenty five years understanding the mechanism of genome protection. J Physiol Biochem.

[19] Lane DP (2005). Cancer. p53, guardian of the genome. Nature.

[20] Orsted DD, Bojesen SE, Tybjaerg-Hansen A, Nordestgaard BG (2007). Tumor supressor p53 Arg72Pro polymorphism and longevity, cancer survival, and risk of cancer in the general population. J Exp Med.

[21] Shu K, Li B, Wu LX (2007). The p53 network: p53 and its downstream genes. Colloids Surf. B Biointerfaces.

[22] Petitjean A, Mathe E, Kato S, Ishioka C, Tavtigian SV, Hainaut P, Olivier M (2007). Impact of mutant p53 functional properties on TP53 mutation patterns and tumor phenotype: lessons from recent developments in the IARC TP53 database. Hum Mutat.

[23] Thomas M, Kalita A, Labrecque S, Pim D, Banks L, Matlashewki G (2001). Two polymorphic forms of wild type p53 differ biochemically and biologically. Mol Cell Biol.

[24] Chosdol K, Ahuja A, Rathore A (2002). Study of p53 codon 72 polymorphism in various etnic groups of North India. Curr Sci.

[25] Vijayaraman KP, Veluchamy M, Murugesan P (2012). p53 Exon 4 (codon 72) Polymorphism and Exon 7 (codon 249) Mutation in Breast Cancer Patients in Southern Region (Madurai) of Tamil Nadu. Asian Pacific J Cancer Prev.

[26] Damin APS, Frazzon APG, Damin DC (2006). Evidence for an association of TP53 codon 72 polymorphism with breast cancer risk. Cancer Detect Prev.

[27] Pharoah PD, Day NE, Caldas C (1999). Somatic mutations in the p53 gene and prognosis in breast cancer: a meta-analysis. Br J Cancer.

[28] Thomas M, Kalita A, Labrecque S, Pim D, Banks L, Matlashewki G (1999). Two polymorphic forms of wild type p53 differ biochemically and biologically. Mol Cell Biol.

[29] Kimbi GC, Kew MC, Yu MC, Arakawa K, Hodkinson J (2005). 249ser p53 mutation in the serum of black southern African patients with hepatocellular carcinoma. J Gastroenterol Hepatol.

[30] Hussain SP, Amstad PR (2001). Mutability of p53 hotspot codons to benzo(a)pyrene diol epoxide (BPDE) and the frequency of p53mutations in nontumorous human lung. Cancer Res.

[31] Sullivan A, Syed N, Gasco M, Bergamaschi D, Trigiante G, Attard M, Farrell PJ, Smith P, Lu X, Crook T (2004). Polymorphism in wild-type p53 modulates response to chemotherapy in vitro and in vivo. Oncogene.

[32] Tommiska J, Eerola H, Heinonen M, Salonen L, Kaare M, Tallila J, Ristimäki K, Von Smitten K (2005). Breast cancer patients with p53Pro72 homozygous genotype have a poorer survival. Clin Cancer Res.

[33] Zhuo W, Zhang Y, Xiang Z, Cai L, Chen Z (2009). Polymorphisms of TP53 codon 72 with breast carcinoma risk: evidence from 12226 cases and 10782 controls. J Exp Clin Cancer Res.

[34] Nelson HH, Wilkjomen M, Marsit CJ, Keisey KT (2005). TP53 mutation, allelism and survival in non-small cell lung cancer. Carcinogenesis.

[35] Shen H, Liu Z, Strom SS, Spitz MR, Lee JE, Gershenwald JE, Ross MI, Mansfield PF (2003). p53 codon 72 Arg homozygotes are associated with an increased risk of cutaneous melanoma. J Invest Dermatol.

[36] Costa KA, Guillo LA (2012). TP53 codon 72 polymorphism in pigmentary phenotypes. J Biosci.

[37] Kalemi TG, Lambropoulos AF, Gueorguiev M (2005). The association of p53 mutations and p53 codon 72, Her 2 codon 655 and MTHFR C677T polymorphisms with breast cancer in Northern Greece. Cancer Let.

[38] Papadakis EN, Dokianakis DN, Spandidos DA (2000). p53 codon 72 polymorphism as a risk factor in the development of breast cancer. Mol Cell Biol Res Commun.

[39] Alawadi S, Ghabreau L, Alsaleh M, Abdulaziz Z, Rafeek M, Akil N, Alkhalaf M (2011). P53 gene polymorphisms and breast cancer risk in Arab women. Med Oncol.

